# Forest bathing improves inflammatory markers, SpO_2_, and subjective symptoms related to chronic obstructive pulmonary disease (COPD) in male subjects at risk of developing COPD

**DOI:** 10.1093/joccuh/uiaf041

**Published:** 2025-07-17

**Authors:** Qing Li, Norimasa Takayama, Yukako Kimura, Hiroshi Takayama, Shigeyoshi Kumeda, Takashi Miura, Tsunemi Kitagawa, Yoichiro Aoyagi, Michiko Imai

**Affiliations:** Department of Rehabilitation Medicine, Graduate School of Medicine, Nippon Medical School, Tokyo, Japan; Forestry and Forest Products Research Institute, Forest Research and Management Organization, Tsukuba, Japan; itswellness org, Tokyo, Japan; Takayama Orthopedic Clinic, Tokyo, Japan; Nagano Prefectural Kiso Hospital, Nagano Prefecture, Japan; Agematsu Town Office, Nagano Prefecture, Japan; Department of Rehabilitation Medicine, Graduate School of Medicine, Nippon Medical School, Tokyo, Japan; Department of Rehabilitation Medicine, Graduate School of Medicine, Nippon Medical School, Tokyo, Japan; INFOM (International Society of Nature and Forest Medicine), Tokyo, Japan

**Keywords:** α1-antitrypsin (α1-AT), chronic obstructive pulmonary disease (COPD), C-reactive protein (CRP), fibrinogen, forest bathing, interleukin 6 (IL-6)

## Abstract

**Background:**

Chronic obstructive pulmonary disease (COPD) is the fourth leading cause of death worldwide, responsible for 3.5 million deaths in 2021. Effective preventive measures are needed. Forest bathing has been reported to have positive effects on the immune system. In addition, the clean air, mild climate, phytoncides, high oxygen concentration, and other elements of forests are expected to have benefits for respiratory diseases such as COPD. Based on the above background, this study used a randomized crossover design to examine the improving effects of forest bathing on inflammatory markers and subjective symptoms related to COPD.

**Methods:**

Thirty male subjects aged 63.1 ± 7.5 years were recruited after obtaining informed consent. These subjects participated in day trips to a Japanese cypress forest park and to a city area of Nagano Prefecture as a control in June 2024. Blood samples were taken in the afternoon of each day before and after the walks. Concentrations of α1-antitrypsin (α1-AT), C-reactive protein (CRP), fibrinogen, and interleukin 6 (IL-6) in blood were measured. Percutaneous oxygen saturation (SpO_2_), the profile of mood states (POMS), and questionnaires for subjective fatigue and respiratory symptoms and sleep quality were carried out before and after each trip.

**Results:**

Forest bathing significantly decreased the concentrations of blood CRP, α1-AT, IL-6, and fibrinogen, significantly increased SpO_2_, reduced subjective fatigue and respiratory symptoms, improved sleep and the scores of positive feelings, and reduced the scores for negative emotions in POMS.

**Conclusions:**

Forest bathing may improve inflammatory markers, SpO_2_, and subjective symptoms related to COPD.

## Teaser

Key points

- It has been reported that forest bathing has benefits for immune function; however, there is insufficient evidence about the effects of forest bathing on inflammatory markers related to COPD.
- This study found that forest bathing improves inflammatory markers related to COPD, suggesting preventive effects for COPD.

## 1. Introduction

According to a World Health Organization report, chronic obstructive pulmonary disease (COPD) is the fourth leading cause of death worldwide, responsible for 3.5 million deaths in 2021, or approximately 5% of all global deaths (first is ischemic heart disease, second is COVID-19, third is stroke).^1^^,^^2^ In Japan, the results of the large-scale epidemiological survey named the Nippon COPD Epidemiology study conducted by Fukuchi et al^3^ showed that the prevalence of COPD in Japanese people aged 40 years or older is 8.6%, with an estimated 5.3 million patients, and that the prevalence of COPD in Japanese people tends to increase with age. It has been reported that the immune system and inflammatory markers such as certain cytokines play an important role in the mechanism of COPD.^4^^-^^10^ Thomsen et al^4^ reported that blood fibrinogen levels increased with the severity of COPD, fibrinogen correlated with an increased risk of COPD exacerbation, and this association was also observed in patients with mild COPD and COPD without a history of exacerbation. In addition, Fermont et al^6^ conducted a meta-analysis in which they extracted and synthesized 61 studies, calculated pooled hazard ratios, and evaluated the association between various biomarkers and clinical outcomes in patients with stable COPD. A decrease in 6-minute walk distance and an increase in heart rate and fibrinogen were associated with an increased risk of death. In addition, a decrease in 6-minute walk distance and an increase in fibrinogen were also associated with exacerbations. Higashimoto et al^7^^,^^8^ reported that serum α1-antitrypsin (α1-AT) and interleukin 6 (IL-6) levels were significantly higher in COPD patients than in control subjects. Recently, Ellingsen et al^9^ conducted an identifying exacerbations cohort study of COPD and found that C-reactive protein (CRP), fibrinogen, and white blood cells (WBCs), assessed during stable-phase COPD, enhanced acute exacerbation prediction in COPD. Aslani et al^10^ also found that IL-6 serum levels increased in COPD patients.

**Table 1:** Basic information about the participants in groups A and B.

	**Group A (*n* = 15)**		**Group B (*n* = 15)**		
	**Mean**	**SD**	**Mean**	**SD**	***P* level** ^**a**^
Age, y	62.6	8.0	63.5	7.1	>0.05
COPD score	5.5	1.4	5.6	1.5	>0.05
Smoking^b^	13		11		>0.05
Brinkman Index (BI)	391.3	320.0	368.3	395.0	>0.05
Sleep time, h	6.4	0.6	6.5	1.0	>0.05
Daily exercise habits^b^	11		14		>0.05
Alcohol^b^	12		11		>0.05
COVID-19^b^	7		10		>0.05
SBP, mmHg	126.7	15.1	123.6	13.7	>0.05
DBP, mmHg	78.8	11.9	78.3	11.2	>0.05
Pulse rate, beats/min	72.0	10.9	72.0	9.7	>0.05
Height, cm	172.3	5.9	171.5	4.7	>0.05
BW, kg	70.8	7.8	73.1	9.5	>0.05
Body mass index, kg/m^2^	23.9	3.2	24.8	2.7	>0.05
Pollen allergies^b^	5		6		>0.05
Hypertension^b^	6		5		>0.05
Diabetes^b^	3		3		>0.05

Abbreviations: BW, body weight; COPD, chronic obstructive pulmonary disease; DBP, diastolic blood pressure; SBP, systolic blood pressure.

a Items marked with b were statistically analyzed using a chi-square test and others were statistically analyzed using a 2-sample *t* test with equal variances.

b Number of participants with this condition or habit.

Forest bathing, also known as Shinrin-yoku, is a Japanese practice that involves immersing oneself in a forest environment to promote physical and mental well-being. Forest bathing has been reported to have a positive effect on the immune system and suppress inflammatory responses caused by cytokines.^11^^-^^14^ In addition, the clean air, mild climate, phytoncides (aroma),^11^^,^^13^ high oxygen concentration, and other elements of forest environments and the relaxing effects of forest bathing^11^^-^^20^ are expected to have an improving effect on respiratory diseases such as COPD. Jia et al^14^ found that forest bathing showed benefits for elderly patients with COPD by decreasing levels of proinflammatory cytokines including IL-6, IL-8, interferon-γ, IL-1β, and CRP (all *P* < .05). However, this study did not use a randomized crossover research design and the subjects were only 10 in the forest bathing group and only 8 in the city walking group.^14^ Hence there are concerns about bias due to the small numbers of participants and inappropriate experimental design. Against this background, the present study used a randomized crossover design to examine the improving effects of forest bathing on inflammatory markers and subjective symptoms related to COPD in 30 male subjects.

It has been reported that occupational exposure to chemicals contributes to the development and progression of COPD. Therefore, the study is relevant in the field of occupational health promotion.^21^^,^^22^

## 2. Subjects

This study was conducted on 30 male subjects aged 46 to 75 years (Mean 63.1 ± 7.5 years) who scored a total of 4 points or more on the COPD-PS (population screener) GOLD questionnaire^23^^,^^24^ (see Supplementary Material 1) and were considered at risk of developing COPD. The 30 subjects were randomly divided into groups A and B (Table 1). There were no significant differences between groups A and B in terms of age, COPD scores, lifestyle habits for cigarette smoking, sleeping hours, daily physical exercise, alcohol consumption, blood pressure, pulse rate, height, body weight, or body mass index. Written informed consent was obtained from all subjects after a full explanation of the study procedures. All subjects stayed in the same hotel and ate the same meals. To eliminate the impact of alcohol, the subjects abstained from alcohol during the experiment. This experiment was carried out in accordance with the Declaration of Helsinki, and was approved by Nippon Medical School Central Ethics Committee.

**Table 2:** Walking time, distance, and speed for forest and city walks of the guides.

		**Forest area**		**City area**	
		**AM**	**PM**	**AM**	**PM**
June 22 (Day 1)	Time	09:59-11:30	12:45-14:15	10:00-11:26	12:54-14:21
	Walking time	1:31:11	1:30:27	1:26:23	1:26:43
	Distance, km	2.3	2.3	2.4	2.4
	Speed, km/h	1.5	1.5	1.7	1.7
June 23 (Day 2)	Time	09:59-11:31	12:45-14:15	10:00-11:27	12:45-14:13
	Walking time	1:32:34	1:30:22	1:27:44	1:28:18
	Distance, km	2.7	2.5	2.4	2.4
	Speed, km/h	1.7	1.7	1.6	1.6

## 3. Methods

### 3.1. Experimental fields

The experimental site for forest bathing in this study was the Akasawa Natural Recreation Forest, and the control site for comparison was Ina City, Nagano Prefecture, as reported previously.^15^^,^^16^ Akasawa Natural Recreation Forest is home to the Kiso Five Trees (Hinoki cypress: Chamaecyparis obtuse, Sawara cypress: Chamaecyparis pisifera, Nezuko cypress: Thuja standishii, Asunaro cypress: Thujopsis dolabrata, Koyamaki, or Japanese umbrella pine: Sciadopitys verticillata), mainly Kiso Hinoki, as well as other trees such as Shiromoji, Marubanoki, and Kousa dogwood. The area is 93.5% forested, and is said to be among the 3 most beautiful forests in Japan. However, there are almost no trees on the walking trails in Ina City.^16^ As previously reported, phytoncides such as alpha-pinene and beta-pinene were detected in the ambient air of Akasawa Natural Recreational Forest, the site of the forest bathing experiment, but no phytoncides were detected in the ambient air of the city.^17^ On the other hand, air pollutants such as toluene, benzene, acetone, and xylene were detected in urban ambient air but not in forest ambient air.^25^

### 3.2. Experimental design and forest and urban walks

This study used a non-blind, randomized, parallel-group comparative study design (randomized crossover design).^15^^,^^16^ Participants were randomized by age and divided into 2 groups. The schedule was 3 days/2 nights in total (Supplementary Material 2). The detailed schedule was as follows.

#### 3.2.1. Day 1

Departure from Tokyo in the morning, arrival at Kiso Prefectural Hospital in Nagano Prefecture in the afternoon. Questionnaire surveys were conducted before blood sampling, which was done at 4:00 pm. Then all subjects stayed at the same hotel and ate the same meals.

#### 3.2.2. Day 2

Group A was randomly assigned to the city site and group B was assigned to the forest site. Questionnaire surveys were conducted before and after forest bathing or the city walk. Blood was sampled at Kiso Prefectural Hospital at 4:00 pm, then all subjects stayed at the same hotel and ate the same meals.

#### 3.2.3. Day 3

On the third day, the subjects switched field sites. Questionnaire surveys were conducted before and after forest bathing or city walk. Blood was sampled at Kiso Prefectural Hospital at 4:00 pm, then all subjects returned home.

Information about walking time, distance, and speed in the forest park and city area are shown in Table 2. The walking time, distance, and speed of the guides who guided the participants were measured and used as the amount of walking exercise of the subjects. As shown in Table 2, no significant differences were found between walking time, distance, and speed of the guides between the urban and forest areas. The forest bathing walk was conducted under the guidance of a forest therapist certified by the NPO Forest Therapy Society. During the walk, the subjects walked slowly in a line guided by the forest therapist. They took 2 breaks along the way, did not talk to each other during the walk, and stopped occasionally to enjoy the forest environment using their 5 senses and deep breathing, following the guide. They were instructed to wear comfortable clothing and shoes for the walk. As with the forest walk, the urban walk also involved walking slowly in a line guided by a forest therapist. The participants took 2 breaks along the way, and were instructed not to talk to each other during the walk.

### 3.3 Blood tests, SpO_2_and questionnaire surveys

1. Blood lactate concentration (evaluation of physical activity): blood lactic acid concentration was used to objectively evaluate the amount of exercise and physical activity and measured by Kiso hospital as described previously.^15^^,^^16^
2. Blood fibrinogen, CRP concentrations, and WBCs: blood fibrinogen, CRP, and WBCs are biomarkers for evaluating COPD^4^^,^^6^^,^^9^ and were measured by Kiso hospital.
3. Blood IL-6 concentration: blood IL-6 is also a biomarker for evaluating COPD^7^^,^^8^ and was measured by the Bio Medical Laboratories, Inc in Tokyo, Japan (BML) with an electrochemiluminescence immunoassay.^26^
4. Blood α1-AT concentration: blood α1-AT is another biomarker for evaluating COPD^7^^,^^8^ and was measured by the BML with nephelometry.^27^
5. Blood tumor necrosis factor α (TNF-α) concentration: blood TNF-α is also a biomarker for COPD and was measured by the BML with an enzyme immunoassay.^28^
6. Percutaneous oxygen saturation (SpO_2_): SpO_2_ is an important parameter to evaluate COPD and was determined by a pulse oximeter.
7. Questionnaire survey of subjective symptoms related to COPD: a COPD assessment test (CAT) was used^29^ (Supplementary Material 3). The CAT is a questionnaire that evaluates the symptoms and condition of COPD patients, but it can also be used by those at risk of developing COPD because it includes questions about cough, phlegm, respiratory symptoms, and sleep.^29^
8. Questionnaire survey on sleep: the MA version of the OSA Sleep Questionnaire has been used to evaluate the effect of forest bathing in both males and females^15^^,^^16^ (Supplementary Material 4).
9. Profile of Mood States (POMS) questionnaire survey: POMS 2 in Japanese was used^15^^,^^16^ (Supplementary Material 5). The POMS 2 test has been applied to evaluate the practice of active rest by workplace units for improving personal relationships, mental health, and physical activity among workers^30^ and the effects of forest bathing.^15^^,^^16^
10. Fatigue subjective symptoms survey: a 30-item questionnaire created by the Fatigue Research Group of the Japan Society for Occupational Health was used (Supplementary Material 6). This questionnaire has been used to evaluate the effect of forest bathing on depression.^16^
11. Measurement of physical activity: the walking time, distance, and speed of the guides who guided the participants were measured.
12. Measurement of environmental temperature, humidity, wind speed, and illuminance:^15^^,^^16^ On the first day of the experiment (June 22), the weather was fine in both the forest and the urban areas. Accompanying the participants in the experiment, we measured the forest environment during the walk every 30 seconds, and the average temperature was 21.1°C, the average relative humidity was 63.3%, the average illuminance on the course was 6526.4 lx, and the wind speed was 0.3 m/s (wind speed was measured at 10 fixed locations along the course). Meanwhile, the urban environment had a temperature of 34.2°C, humidity of 23.4%, illuminance of 56 561.6 lx, and a wind speed of 1.3 m/s. On the second day of the experiment (June 23), the weather was rainy and cloudy in both the forest and the urban settings. The forest environment had a temperature of 17.6°C, humidity of 99.5%, illuminance of 1177.8 lx, and wind speed of 0.2 m/s, whereas the urban environment had a temperature of 22.6°C, humidity of 99.3%, illuminance of 8779.1 lx, and wind speed of 0.5 m/s.

### 3.4. Statistical analysis

When samples are of equal variance, a paired *t* test can be used. In this study, we performed a variance test (*F* test) before performing a *t* test. We confirmed that the data were of equal variance, and so a paired *t* test was used to compare the differences between forest bathing and city walking or between before and after the forest bathing and city walking. A chi-squared test and a 2-sample *t* test with equal variances were used in Table 1. Analyses were performed with the Microsoft Excel software package for Windows.^15^^,^^16^ The significance level for *P* values was set at <.05.

## 4. Results

### 4.1. Effect of walking on blood lactate concentration

Because exercise increases blood lactate, blood lactate concentration is an index that objectively evaluates the amount of exercise and physical activity.^31^ As shown in Table 3, there was no statistically significant difference between urban and forested areas. This also means that there was no statistically significant difference between the amount of exercise during walking in urban and forested areas. The normal value of blood lactate is 5 to 20 mg/dL.

**Table 3:** Effects of forest bathing on lactic acid, fibrinogen, CRP, IL-6, α1-AT, TNF-α, and WBCs in blood.

	**Before** ^**a**^	**After city walking** ^**b**^	**After forest bathing** ^**b**^
Lactic acid, mg/dL	6.37 ± 0.61	6.77 ± 0.46	6.65 ± 0.51
Fibrinogen, mg/dL	287.73 ± 11.54	271.03 ± 9.31^**^	270.60 ± 9.68^**^
CRP, mg/dL	0.14 ± 0.03	0.11 ± 0.03	0.10 ± 0.02^*^
IL-6, pg/mL	3.11 ± 1.13	2.91 ± 0.92	2.37 ± 0.68†
α1-AT, mg/dL	135.2 ± 3.56	143.47 ± 4.12	131.00 ± 3.53††
TNF-α, pg/mL	1.05 ± 0.48	0.99 ± 0.44	1.09 ± 0.43
WBC, ×10^3^/μL	6.56 ± 0.42	6.20 ± 0.39	6.27 ± 0.40

Abbreviations: CRP, C-reactive protein; IL-6, interleukin 6; TNF-α, tumor necrosis factor α; WBC, white blood cells; α1-AT, alpha-1 anti-trypsin.

a “Before” means before walking in the city area or before the forest bathing on June 21.

b ^*^ *P* < .05, ^**^P < .01 compared with before; †*P* < .05, ††*P* < .01 for forest bathing versus city walking by paired *t* test (mean ± SE, *n* = 30).

### 4.2. Effect of forest bathing on blood fibrinogen

As shown in Table 3, after the walks in the city and forest, both values of blood fibrinogen were significantly lower than before. There was no statistically significant difference in the blood fibrinogen concentrations after the walks in the city and forest. This suggests that the walk (exercise) may lower the blood fibrinogen concentration. The standard value for blood fibrinogen concentration is 200-400 mg/dL.

### 4.3. Effect of forest bathing on blood CRP concentration

Blood CRP concentrations after the walks are shown in Table 3. After the walk in the urban and forest areas, both showed lower values than before, but after the walk in the forest area, there was a statistically significant decrease, suggesting a decrease in CRP due to forest bathing. The standard value for CRP is 0-0.14 mg/dL.

### 4.4. Effect of forest bathing on blood high-sensitivity IL-6

Blood high-sensitivity IL-6 concentration after the walks is shown in Table 3. After a forest walk, there was a statistically significant decrease compared with after a walk in the city, suggesting that forest bathing decreased IL-6. The reference value for IL-6 is less than 2.60 pg/mL.

### 4.5. Effect of forest bathing on blood α1-AT

The blood α1-AT concentration after the walks is shown in Table 3. It was significantly lower after the forest bathing compared with after the urban walk, suggesting that a decrease in blood α1-AT concentration was due to forest bathing. The standard value for blood α1-AT concentration is 94-150 mg/dL.

### 4.6. Effect of forest bathing on blood TNF-α concentration

The effect of forest and urban walks on blood TNF-α concentration is shown in Table 3. There was no statistically significant difference before/after or between the urban and forest walks.

### 4.7. Effect of forest bathing on blood WBCs

The effect of forest and urban walks on blood WBCs is shown in Table 3. There was no statistically significant difference before/after or between the urban and forest walks.

### 4.8. Effect of forest bathing on SpO_2_

The effect of forest bathing and urban walks on SpO_2_ is shown in Table 4. After forest bathing the SpO_2_ was significantly higher than before forest bathing and after the city walk suggesting that an improvement in SpO_2_ was due to forest bathing.

**Table 4:** Effect of forest bathing and city walking on SpO_2_, scores of respiratory symptoms related to COPD, and subjective sleep quality.

	**Forest bathing (mean ± SE)**		**City walking (mean ± SE)**	
	**Before**	**After** ^**a**^	**Before**	**After**
SpO_2_, %	96.4 ± 0.29	97.1 ± 0.27^**^††	95.8 ± 0.47	96.0 ± 0.34
Scores of respiratory symptoms	10.0 ± 1.1	7.5 ± 1.2^**^	9.2 ± 1.1	8.8 ± 1.3
Subjective sleep quality^b^				
Factor I	49.87 ± 2.36	55.44 ± 1.98^*^	49.33 ± 2.37	50.84 ± 2.45
Factor II	43.64 ± 2.39	43.93 ± 2.91	40.87 ± 3.08	44.89 ± 2.47
Factor III	43.60 ± 3.02	47.88 ± 2.77^*^	45.09 ± 3.33	43.65 ± 3.24
Factor IV	47.43 ± 1.61	53.74 ± 2.29^*^	46.26 ± 2.56	50.41 ± 2.68
Factor V	48.29 ± 1.99	53.70 ± 2.25^*^	44.40 ± 3.02	49.91 ± 2.84

Abbreviation: SpO_2_, percutaneous oxygen saturation.

a ^*^ *P* < .05, ^**^P < .01 compared with before; ††*P* < .01 for forest bathing versus city walking by paired *t* test; *n* = 30 for SpO_2_ (%) and scores of respiratory symptoms; *n* = 15 for subjective sleep quality.

b Subjective sleep quality: Factor I: sleepiness on rising; Factor II: initiation and maintenance of sleep; Factor III: frequent dreaming; Factor IV: feeling refreshed (recovery from fatigue); Factor V: sleep length.

**Table 5:** Effect of forest bathing on scores in the POMS test (mean ± SE).^a^

	**AH**	**CB**	**DD**	**FI**	**TA**	**VA**	**F**	**TMD**
Before^b^	2.7 ± 0.6	3.2 ± 0.6	3.0 ± 0.6	4.7 ± 0.7	4.3 ± 0.7	10.0 ± 0.8	11.0 ± 0.7	7.7 ± 2.9
City^c^	1.7 ± 0.6^*^	1.7 ± 0.5^**^	2.3 ± 0.8	4.8 ± 0.7	2.5 ± 0.7^**^	9.9 ± 0.9	10.8 ± 0.9	3.2 ± 3.2
Forest^d^	1.6 ± 0.5^*^	1.8 ± 0.4^**^	1.4 ± 0.4^**^	4.2 ± 0.7	2.7 ± 0.5^**^	11.0 ± 0.9†	11.4 ± 0.9	1.0 ± 2.3^**^
*n*	30	30	30	30	30	30	30	30

a ^*^ *P* < .05, ^**^P < .01 compared with before; †*P* < .05 for forest bathing versus city walking by paired *t* test (mean + SE).

b “Before” means before walking in the city area or before the forest bathing on June 16.

c “City” means after walking in the city area.

d “Forest” means after walking in the forest (forest bathing).

Abbreviations: AH, anger-hostility; CB, confusion-bewilderment; DD, depression-dejection; F, friendliness; FI, fatigue-inertia; POMS, profile of mood states; TA, tension-anxiety; TMD, total mood disturbance; VA, vigor-activity.

### 4.9. Effect of forest bathing on respiratory symptoms related to COPD

The effect of forest bathing and urban walks on respiratory symptoms is shown in Table 4. The respiratory symptoms related to COPD after forest bathing were significantly decreased compared with before. On the other hand, there was no significant difference between before and after the city walk, suggesting that forest bathing improves respiratory symptoms related to COPD.

### 4.10. Effects of forest bathing and urban walks on sleep

The effects of forest bathing and city walks on sleep are shown in Table 4. Compared with before forest bathing, sleepiness upon waking (factor I), dreaming (factor III), fatigue recovery (factor IV), and sleep time (factor V) were significantly improved after forest bathing. Overall, forest bathing was found to improve sleep quality. Compared with before the city walk, sleepiness upon waking (factor I), falling asleep and maintaining sleep (factor II), fatigue recovery (factor IV), and sleep duration (factor V) after the city walk showed a tendency to improve, but the difference was not statistically significant. However, after the city walk, dreaminess (factor III) showed a tendency to worsen, but the difference was not statistically significant. Overall, no improvement in sleep quality was observed in the city walk.

### 4.11. Effects of forest bathing on POMS scores

The effects of forest bathing and urban walk on POMS scores are shown in Table 5. It was found that AH (anger-hostility), CB (confusion-bewilderment), DD (depression-dejection), TA (tension-anxiety), and TMD score (total score) were all significantly improved after forest bathing compared with before (*P* < .05 to <.01). Furthermore, VA (liveliness-vitality) also improved significantly after forest bathing compared with post-city walk, confirming the effect of forest bathing (*P* < .05). AH (anger-hostility), CB (confusion-bewilderment), and TA (tension-anxiety) also improved significantly after the city walk compared with pre-walk values. Taken together, forest bathing significantly increased vitality, improved negative emotions, and demonstrated a relaxing effect.

### 4.12. Effect of forest bathing on fatigue symptom scores

Table 6 shows the change in fatigue symptom scores before and after forest bathing and the urban walk. Group I evaluates drowsiness and lethargy (reduced vitality), Group II evaluates difficulty in concentrating (reduced energy), and Group III evaluates physical discomfort on the scale of fatigue symptoms. Compared to the pre-values, the scores of Groups I, II, and III all significantly decreased after forest bathing. Moreover, forest bathing significantly decreased the scores of Groups I and III compared with the urban walk, demonstrating the effect of forest bathing in improving fatigue. It was found that Group II’s score after both the forest and urban walk significantly decreased compared with the pre-values, but the difference between the forest bathing and urban walk was not significant.

**Table 6:** Effect of forest bathing on subjective fatigue scores (mean ± SE).^a^

**Group** ^**b**^	**Before** ^**c**^	**After city walking**	**After forest bathing**
Group I	3.50 ± 0.55	3.13 ± 0.57	2.10 ± 0.37^**^†
Group II	3.10 ± 0.49	1.30 ± 0.44^**^	1.03 ± 0.31^**^
Group III	2.27 ± 0.35	2.10 ± 0.49	0.93 ± 0.24^**^†
*n*	30	30	30

a ^**^ *P* < .01 compared with before; †*P* < .05 for forest bathing versus city walking by paired *t* test.

b Group I evaluates drowsiness and lethargy (reduced vitality), Group II evaluates difficulty in concentrating (reduced energy), and Group III evaluates physical discomfort on the scale of fatigue symptoms.

c “Before” means before walking in the city area or before forest bathing.

## 5. Discussion

To date, there has only been 1 study of the effects of forest bathing in COPD patients. However, there are concerns about bias due to the small number of participants and inappropriate experimental design of that study.^14^ Against this background, the present study used a randomized crossover design to examine the effects of forest bathing on 30 male subjects at risk of developing COPD. Blood CRP, WBCs, α1-AT, IL-6, and fibrinogen have been reported as evaluation indicators of COPD.^4^^-^^10^ We found that forest bathing significantly reduced blood CRP, IL-6, α1-AT, and fibrinogen concentrations, suggesting that forest bathing has an effect of improving inflammation. Jia et al^14^ found that forest bathing showed benefits for elderly patients with COPD by decreasing levels of proinflammatory cytokines including IL-6, IL-8, interferon-γ, IL-1β, and CRP (all *P* < .05). Kim et al^32^ reported that barefoot walking in urban forests reduced CRP levels in healthy adults, thus supporting our results. On the other hand, Mao et al^33^ in 2017 reported that forest bathing reduced the level of IL-6, but not TNF-α and CRP in elderly patients with chronic heart failure. However, in their 2018 study, Mao et al^34^ reported that forest bathing reduced the level of TNF-α, but not IL-6, in elderly patients with chronic heart failure. Their 2 studies showed different results. Based on these results, further research is needed on the effects of forest bathing on CRP, IL-6, and TNF-α.

Decreased SpO_2_ and respiratory symptoms have been reported in COPD patients.^35^ We found that forest bathing significantly increased SpO_2_ in subjects at risk of developing COPD, suggesting an improving effect on SpO_2_. In addition, forest bathing also significantly reduced respiratory symptoms related to COPD, suggesting an improving effect on respiratory symptoms related to COPD.

It has been reported that sleep disorders occur frequently in COPD patients.^36^^,^^37^ Obstructive sleep apnea and insomnia are common in COPD patients.^37^ In the present study, we found that forest bathing significantly improved sleepiness upon waking, dreaminess, fatigue recovery, and sleep time, demonstrating the improving effect of forest bathing on sleep quality. Other studies have also reported the benefits of forest bathing for sleep.^15^^,^^16^^,^^38^^-^^41^

Stress levels have been reported to be high in COPD patients.^42^ It has been reported that depression-related symptoms occur frequently in COPD patients.^43^ Forest bathing reduces stress and stress hormones and improves depression-related symptoms.^11^^-^^20^ This study confirmed that forest bathing significantly increased vitality scores and significantly reduced the total scores of anger-hostility, confusion-embarrassment, tension-anxiety, and bad mood, suggesting an improving effect on depression and a relaxing effect of forest bathing in the POMS test. This suggests that forest bathing may improve mental health and reduce stress in COPD patients. We also found that forest bathing significantly reduced subjective fatigue symptoms, suggesting an improving effect of forest bathing on fatigue in COPD patients. To control for the effects of alcohol, the subjects did not consume alcohol during the study period. To control the effect of physical activity, as shown in Table 2, subjects walked the same distance (4.6-5.2 km/d) during the same period in both trips. We also confirmed that there was no significant difference in lactic acid concentrations in serum between the forest bathing and urban area walking (Table 3).

On the first day of the experiment (June 22), the weather was fine in both the forest and the urban areas. On the second day of the experiment (June 23), the weather was rainy and cloudy in both the forest and the urban areas. The temperature in the forest environment was lower than in the city environment. The humidity in the forest environment on the first day was higher than in the urban environment. These climatic conditions may have a certain effect on the results. However, because this study used a randomized crossover design, the forest bathing group and the urban walking group experienced sunny and rainy weather equally, so the impact of the weather on the experimental results is the same for both groups.^16^ However, since the effects of climatic conditions cannot be denied, we concede them to be limitations of this study.

Although forest bathing is not a direct treatment for COPD, it can be a complementary practice to improve mental well-being, reduce stress, and encourage gentle physical activity. COPD patients should approach forest bathing with caution, ensuring the environment is safe and suitable for their condition.

Why does forest bathing have such effects? Forest bathing involves all the 5 senses—sight, smell, hearing, touch, and taste; all are stimulated by the forest environment with its quiet atmosphere, beautiful scenery, calm climate, pleasant aromas, and clean fresh air compared with city environments.^11^ The olfactory effect of forest scents (phytoncides) from trees is of paramount importance. In experiments we have found that phytoncides from trees boosted immune functions by increasing the activity of human natural killer cells and intracellular anticancer proteins such as perforin, granulysin and granzymes A and B, both *in vitro*^44^ and *in vivo*,^13^ and reducing stress hormones *in vivo*.^13^ In addition, as previously reported, phytoncides such as alpha-pinene and beta-pinene were detected in the ambient air of Akasawa Natural Recreational Forest, the site of the forest bathing experiment, but no such phytoncides were detected in the ambient air of the city.^17^^,^^25^ On the other hand, air pollutants such as toluene, benzene, acetone, and xylene were detected in urban ambient air but not in forest ambient air.^25^

The potential benefits of forest bathing for COPD patients are as follows:

Stress reduction: COPD patients often experience anxiety and stress due to their condition.^39^^,^^40^ Forest bathing has been shown to lower levels of stress hormones such as cortisol, adrenaline, and noradrenaline, reduce stress, and promote relaxation,^11^^-^^20^ which can improve overall quality of life.
Improved mood: Spending time in forests can boost mood and reduce symptoms of depression,^11^^-^^20^ which is common among COPD patients.^42^^,^^43^
Better air quality: Forests typically have cleaner air compared with urban environments.^11^^,^^12^ For COPD patients, breathing in fresh, oxygen-rich air may be beneficial, although this depends on the specific forest environment and air quality.
Mild physical activity: Forest bathing encourages gentle walking and movement,^11^^,^^12^^,^^15^^-^^17^ which can help maintain physical fitness without overexertion. This is important for COPD patients, as regular, moderate exercise can improve lung function and overall health.
Immune system support: Several studies have suggested that forest bathing and phytoncides released by trees may enhance immune function,^11^^-^^13^^,^^17^^,^^44^ although more research is needed to confirm this benefit for COPD patients.

When COPD patients practice forest bathing, the following considerations are necessary:

Air quality: Ensure the forest environment has good air quality, free from pollutants or allergens that could exacerbate COPD symptoms.^44^
Pollen and hay fever: Be cautious during high pollen seasons, as pollen and hay fever can trigger breathing difficulties.^45^ In Japan, pollen allergies (such as cedar pollen allergy) generally occur from January to the end of April, and since this study was conducted in late June, the risk of pollen allergy was very low. In fact, none of the participants in the present study had any symptoms of pollen allergy.
Physical limitations: COPD patients should avoid overexertion and choose trails that are flat and easy to navigate.
Consult a doctor: Always consult with a personal physician before starting any new activity, especially for individuals with chronic respiratory conditions.

## 6. Limitations

The present study has the following limitations:

1. The subjects of this study were participants at risk of developing COPD, but not patients with COPD. Initially, we targeted COPD patients, but ultimately we targeted subjects at risk of developing COPD from the perspective of safety and risk management, considering that travel can worsen symptoms in COPD patients. We are planning to target COPD patients in our next study.
2. This study was a field study, but not a laboratory experiment; therefore, various factors such as differences between forest and urban environments in terms of ambient illuminance, temperature, and humidity may affect the results. However, by using a randomized crossover research design, this study eliminated order bias, improved the accuracy of statistical analysis, and the impact of other factors was also minimized. The effect of forest bathing is the sum of the effects on the 5 senses (sight, smell, hearing, taste, and touch) stimulated by the forest environment, including the quiet atmosphere, beautiful scenery, calm climate, pleasant aromas, and clean fresh air compared with city environments.^11^
3. In this study, it was difficult to predict what effect forest bathing and urban walks would have on each parameter, so we determined statistically the difference between forest bathing and urban walk, and between forest bathing, urban walk, and baseline for each parameter. The repeated *t* test may have incurred type I error. However, we have conducted several studies using the same method (repeated paired *t* tests).^12^^,^^13^^,^^15^^-^^17^ In order to compare these past studies with future studies, we performed the analysis in the same way this time. However, because the possibility of type I error cannot be completely ruled out, this is a limitation of this study.

## 7. Conclusions

Our study indicated that forest bathing showed the following benefits for male subjects at risk of developing COPD: (1) reductions in levels of blood CRP, IL-6, α1-AT, and fibrinogen; (2) improvement in SpO_2_; (3) improvement in respiratory symptoms related to COPD; (4) improvement in subjective sleep quality assessed by the OSA-MA; (5) decreased negative mood and increased positive feeling of vigor in the POMS test; and (6) improvement in subjective mental and physical fatigue symptoms.

Taken together, forest bathing showed improving effects on inflammation markers, SpO_2_, respiratory symptoms related to COPD, mental health, and relaxation in male subjects at risk of developing COPD.

## Supplementary Material

Web_Material_uiaf041

## Data Availability

Data in this study are available from the corresponding author if necessary.
