# Supplementary material for: Forest bathing improves inflammatory markers, SpO2, and subjective symptoms related to chronic obstructive pulmonary disease (COPD) in male subjects at risk of developing COPD
Source: J Occup Health. 2025 Jul 17;67(1):uiaf041. doi: 10.1093/joccuh/uiaf041 (PMC12353587; doi:10.1093/joccuh/uiaf041)
Supplement: Web_Material_uiaf041 [file web_material_uiaf041.zip › Supplementary Materials.docx]

**Supplementary Materials**

1. The COPD-PS_GOLD questionnaire in Japanese


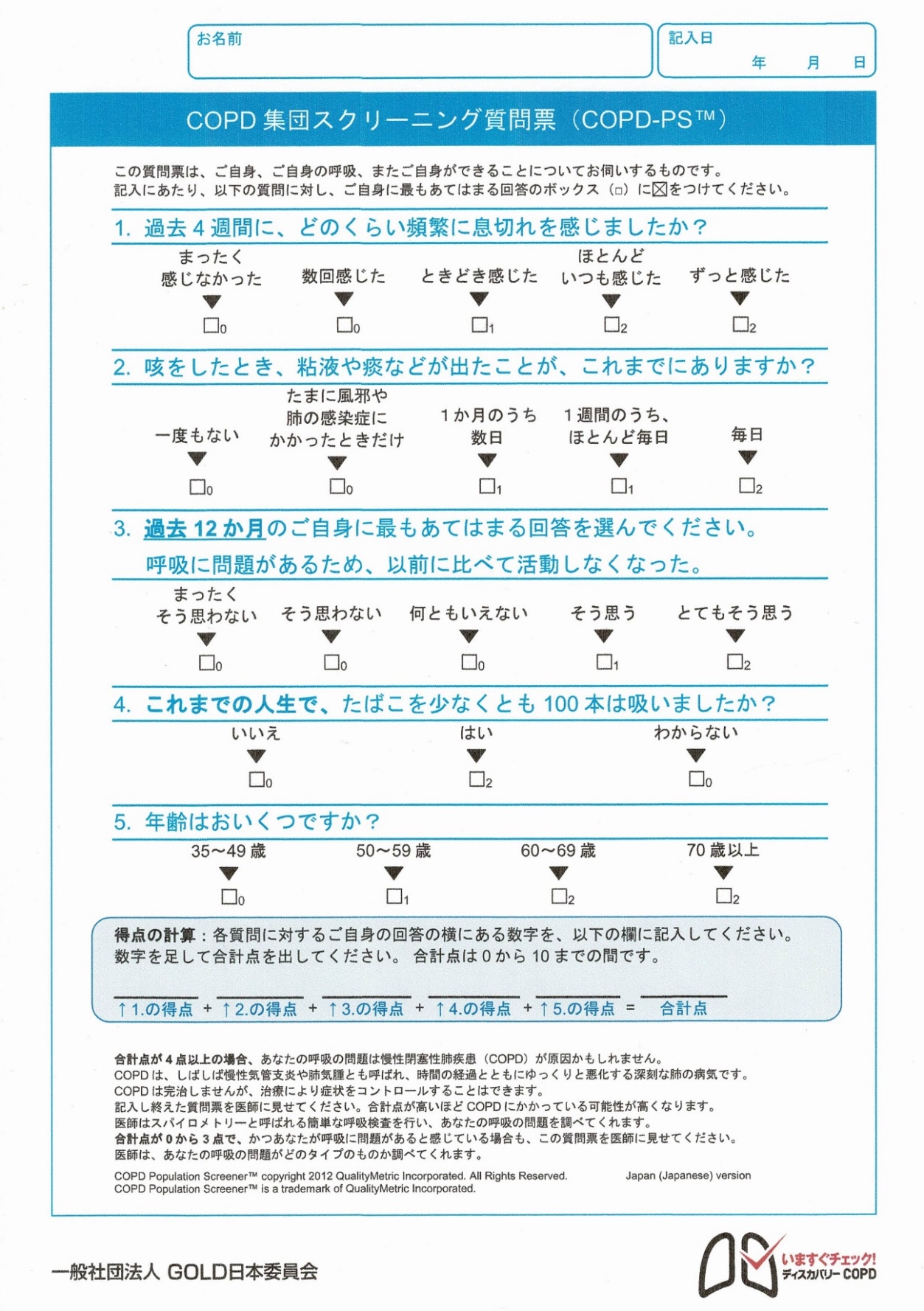


COPD-PS GOLD questionnaire in English

Items

1. During the past 4 weeks, how much of the time did you feel short of breath?

Answers Score

Not of the time 0

A little of the time 0

Some of the time 1

Most of the time 2

All the time 2

1. Do you ever cough up any “stuff,” such as mucus or phlegm?

Answers Score

No, never 0

Only with occasional colds or chest infections 0

Yes, a few days a month 1

Yes, most days a week 1

Yes, every day 2

1. I do less than I used to be a cause of my breathing problems in the past 12 months.

Answers Score

Strongly disagree 0

Disagree 0

Unsure 0

Agree 1

Strongly agree 2

1. Have you smoked at least 100 cigarettes in your ENTIRE LIFE?

Answers Score

No 0

Yes 2

Don't know 0

1. How old are you?

Answers Score

Age 35 to 49 0

Age 50 to 59 1

Age 60 to 69 2

Age 70+ 2

If your total score is 4 or more, your breathing problems may be caused by COPD, meaning you are at risk of developing COPD.

1. The crossover research design


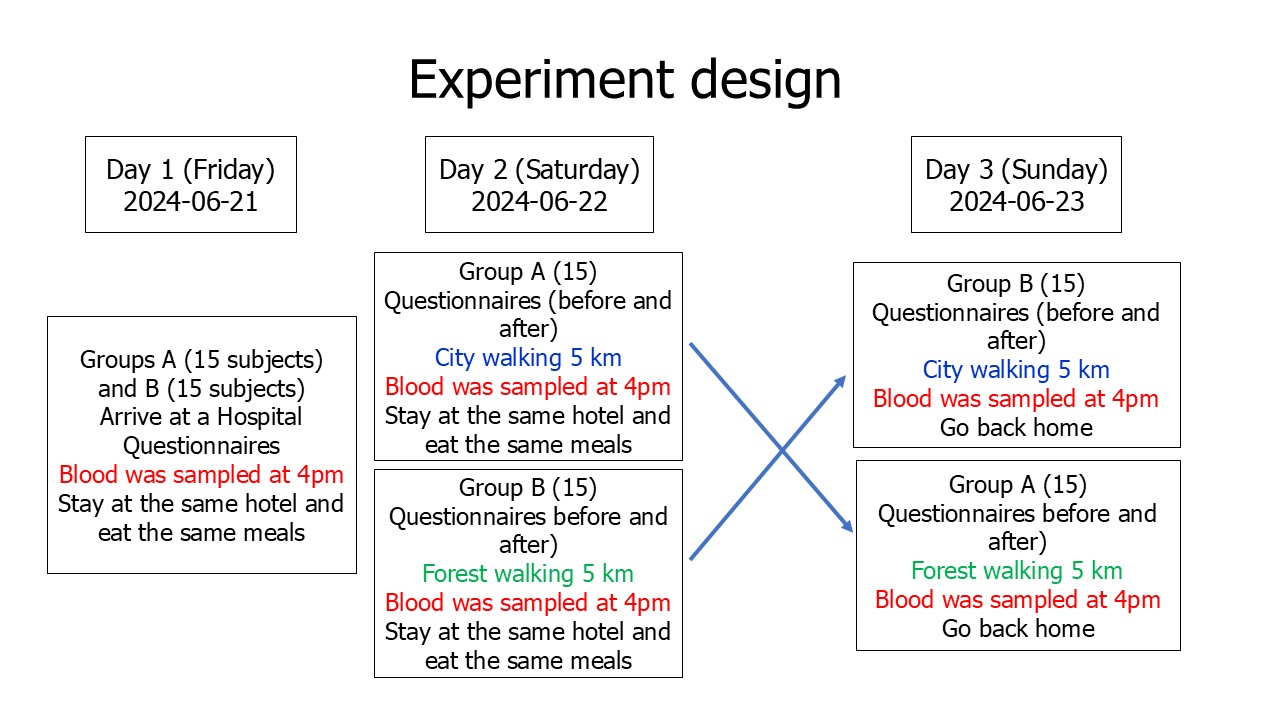


1. Questionnaire survey on subjective symptoms of COPD: CAT (CAT：COPD assessment test）in Japanese


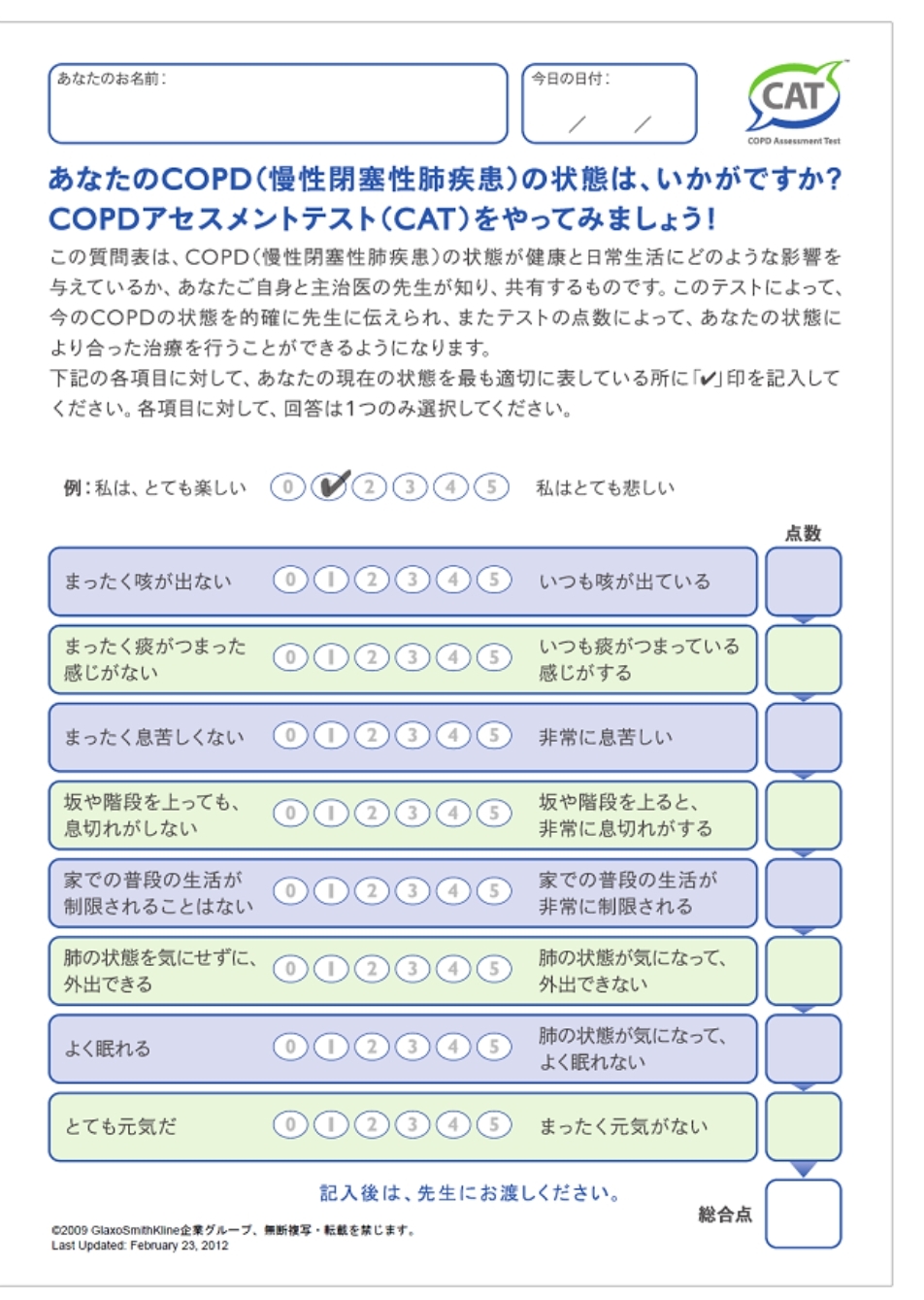


Questionnaire survey on subjective symptoms of COPD: CAT (CAT：COPD assessment test）in English


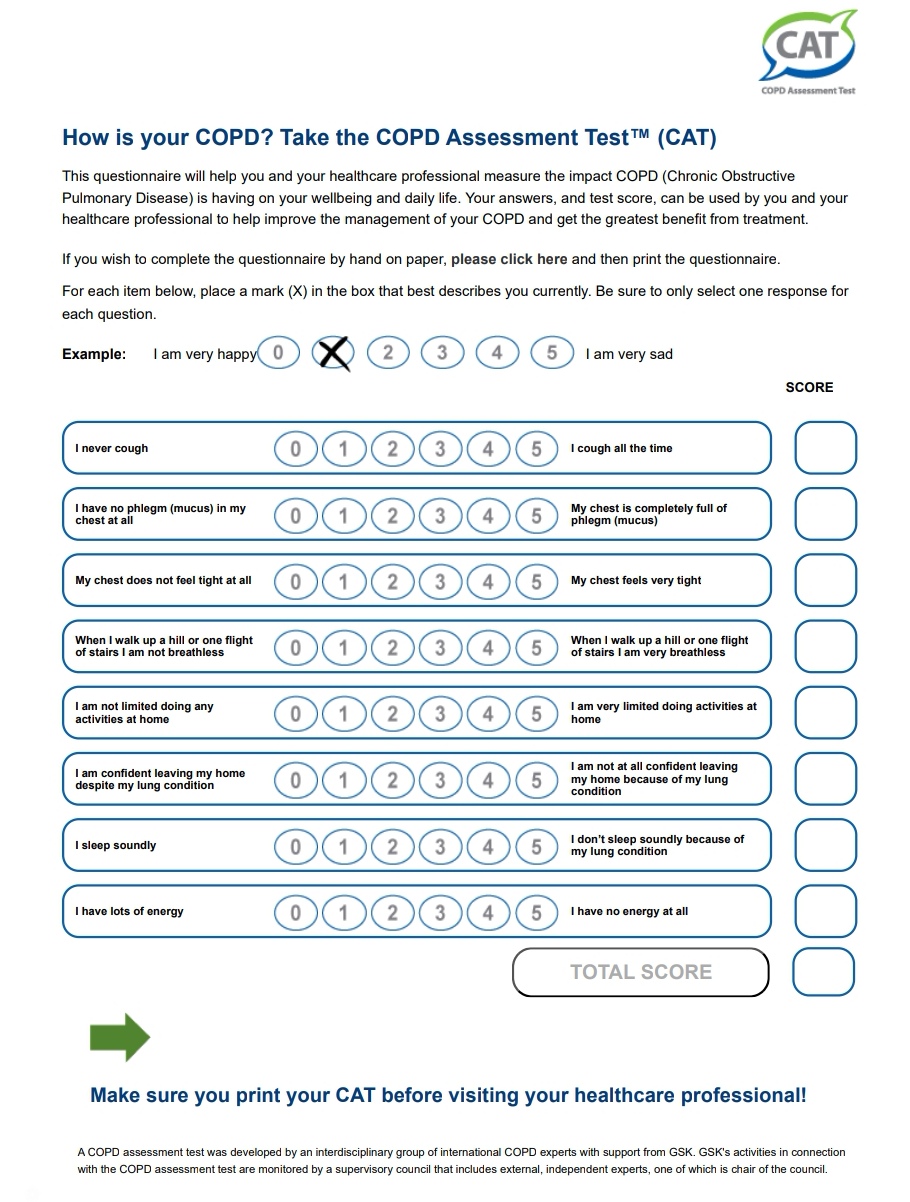


1. Questionnaire survey on sleep: The MA version of the OSA Sleep Questionnaire in Japanese was used

睡眠に関するアンケート調査：OSA睡眠調査票MA版


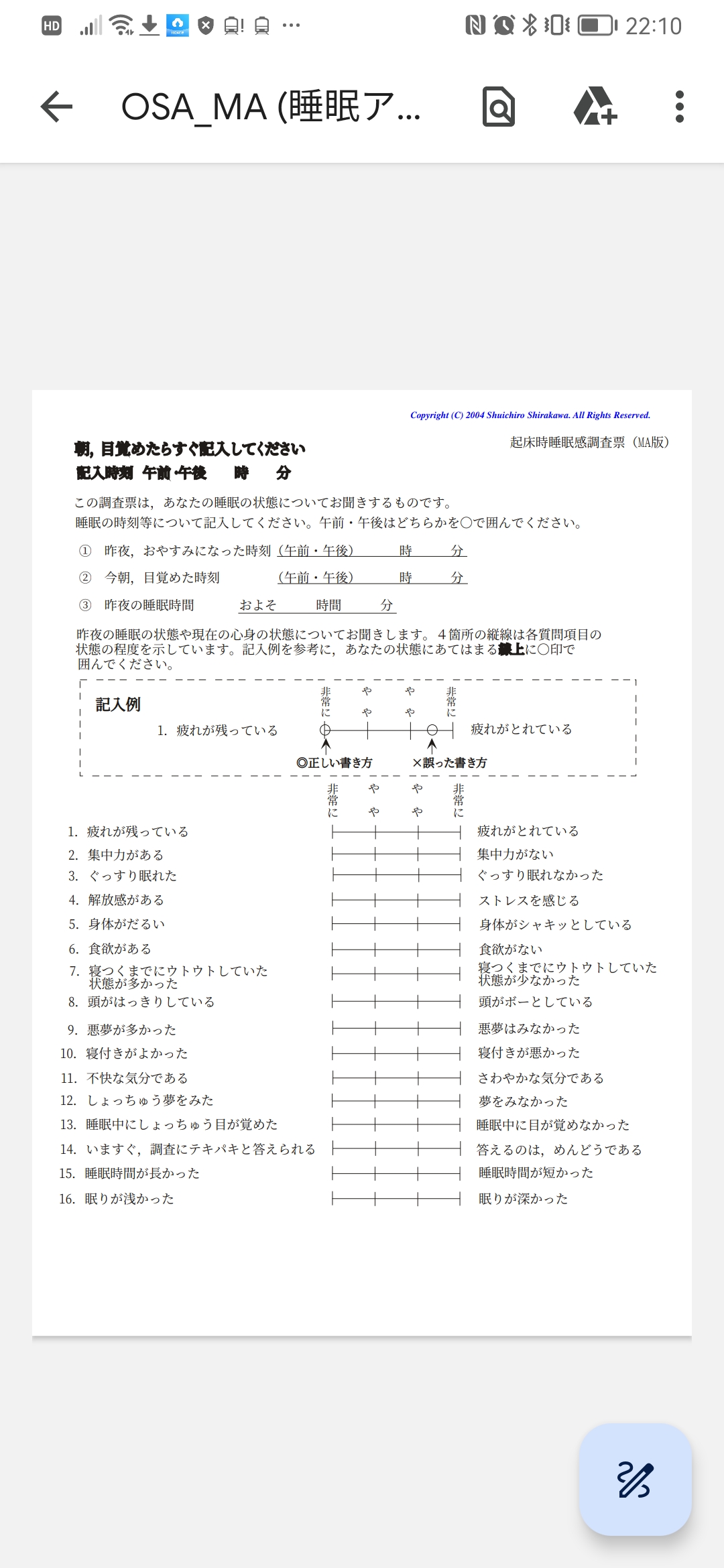


The sleep quality questionnaire using the Ogri-Shirakawa-Azumi sleep inventory MA version (OSA-MA) in English

| No. | Question items | Never | Seldom | Sometimes | Almost always | Factor |
| --- | --- | --- | --- | --- | --- | --- |
| 1 | I still felt tired | 0 | 1 | 2 | 3 | 4 |
| 2 | I couldn't concentrate | 0 | 1 | 2 | 3 | 1 |
| 3 | I couldn't sleep well | 0 | 1 | 2 | 3 | 2 |
| 4 | I felt stressed | 0 | 1 | 2 | 3 | 1 |
| 5 | I felt tired | 0 | 1 | 2 | 3 | 4 |
| 6 | I had no appetite | 0 | 1 | 2 | 3 | 5 |
| 7 | I dozed off a lot | 0 | 1 | 2 | 3 | 2 |
| 8 | I felt dazed | 0 | 1 | 2 | 3 | 1 |
| 9 | I had a lot of nightmares | 0 | 1 | 2 | 3 | 3 |
| 10 | I had trouble falling asleep | 0 | 1 | 2 | 3 | 2 |
| 11 | I felt uncomfortable | 0 | 1 | 2 | 3 | 4 |
| 12 | I had dreams a lot | 0 | 1 | 2 | 3 | 3 |
| 13 | I woke up a lot | 0 | 1 | 2 | 3 | 2 |
| 14 | I was too embarrassed to answer | 0 | 1 | 2 | 3 | 1 |
| 15 | My sleep time was shorter | 0 | 1 | 2 | 3 | 5 |
| 16 | My sleep was shallow | 0 | 1 | 2 | 3 | 2 |

1. POMS2 (Profile of Mood States) in English

POMS 2 of the description of questions, the scale used by the participants to respond to the questions in English

| No. | Questions | Not at all | A little | A fair amount | Quite a lot | Very much |
| --- | --- | --- | --- | --- | --- | --- |
| 1 | I enjoy socializing | 0 | 1 | 2 | 3 | 4 |
| 2 | I feel tense. | 0 | 1 | 2 | 3 | 4 |
| 3 | I feel angry | 0 | 1 | 2 | 3 | 4 |
| 4 | I feel exhausted | 0 | 1 | 2 | 3 | 4 |
| 5 | I feel lively. | 0 | 1 | 2 | 3 | 4 |
| 6 | I feel confused. | 0 | 1 | 2 | 3 | 4 |
| 7 | I care about others. | 0 | 1 | 2 | 3 | 4 |
| 8 | I feel sad. | 0 | 1 | 2 | 3 | 4 |
| 9 | I feel positive. | 0 | 1 | 2 | 3 | 4 |
| 10 | I feel depressed. | 0 | 1 | 2 | 3 | 4 |
| 11 | I feel full of energy | 0 | 1 | 2 | 3 | 4 |
| 12 | I feel confused | 0 | 1 | 2 | 3 | 4 |
| 13 | I feel hopeless | 0 | 1 | 2 | 3 | 4 |
| 14 | I feel anxious | 0 | 1 | 2 | 3 | 4 |
| 15 | I can't concentrate | 0 | 1 | 2 | 3 | 4 |
| 16 | I'm tired | 0 | 1 | 2 | 3 | 4 |
| 17 | I feel like I can be useful to others | 0 | 1 | 2 | 3 | 4 |
| 18 | I feel nervous | 0 | 1 | 2 | 3 | 4 |
| 19 | I feel miserable | 0 | 1 | 2 | 3 | 4 |
| 20 | I can't think clearly | 0 | 1 | 2 | 3 | 4 |
| 21 | I'm exhausted | 0 | 1 | 2 | 3 | 4 |
| 22 | I feel really angry inside | 0 | 1 | 2 | 3 | 4 |
| 23 | I worry about things | 0 | 1 | 2 | 3 | 4 |
| 24 | I can be kind to others | 0 | 1 | 2 | 3 | 4 |
| 25 | I can't do anything myself | 0 | 1 | 2 | 3 | 4 |
| 26 | I feel fed up | 0 | 1 | 2 | 3 | 4 |
| 27 | I feel helpless | 0 | 1 | 2 | 3 | 4 |
| 28 | I feel very angry | 0 | 1 | 2 | 3 | 4 |
| 29 | I trust others | 0 | 1 | 2 | 3 | 4 |
| 30 | I get angry easily | 0 | 1 | 2 | 3 | 4 |
| 31 | I feel worthless | 0 | 1 | 2 | 3 | 4 |
| 32 | I feel energized | 0 | 1 | 2 | 3 | 4 |
| 33 | I'm not sure about things | 0 | 1 | 2 | 3 | 4 |
| 34 | I'm exhausted | 0 | 1 | 2 | 3 | 4 |
| 35 | I'm full of motivation | 0 | 1 | 2 | 3 | 4 |

1. Fatigue subjective symptoms survey: A 30-item questionnaire created by the Fatigue Research Group of the Japan Society for Occupational Health in Japanese was used.

自覚症状記入用紙

ID 氏名 　　日付 令和 年　　月　　日　　時　　分

今の状態についてお聞きします。次の様なことがあったら**○**、なかったら**×**のいずれかを**□**の中にかならずつけてください。

Ⅰ群 Ⅱ群 Ⅲ群

| 1 | 頭が重い |  | 11 | 考えがまとまらない |  | 21 | 頭が痛い |  |
| --- | --- | --- | --- | --- | --- | --- | --- | --- |
| 2 | 全身がだるい |  | 12 | 話をするのがイヤになる |  | 22 | 肩がこる |  |
| 3 | 足がだるい |  | 13 | いらいらする |  | 23 | 腰が痛い |  |
| 4 | あくびが出る |  | 14 | することに間違いが多くなる |  | 24 | 息苦しい |  |
| 5 | 頭がぼんやりする |  | 15 | 気がちる |  | 25 | 口がかわく |  |
| 6 | 眠い |  | 16 | 物事に熱心になれない |  | 26 | 声がかすれる |  |
| 7 | 目が疲れる |  | 17 | ちょっとしたことが思い出せない |  | 27 | めまいがする |  |
| 8 | 動作がぎこちない |  | 18 | 物事が気にかかる |  | 28 | まぶたや筋肉がピクピクする |  |
| 9 | 足元が頼りない |  | 19 | きちんとしていられない |  | 29 | 手足がふるえる |  |
| 10 | 横になりたい |  | 20 | 根気がなくなる |  | 30 | 気分がわるい |  |

Ⅰ群：ねむけとだるさ（活力の低下）

Ⅱ群：注意集中の困難（気力の低下）

Ⅲ群：身体違和感

（ⅠとⅢは身体症状、Ⅱは精神症状とも言える）

昨夜の睡眠時間は　　　時間

Fatigue subjective symptoms survey: A 30-item questionnaire created by the Fatigue Research Group of the Japan Society for Occupational Health in English

Subjective fatigue symptoms questionnaire

Name: 　Date:

I would like to ask you about your current situation. If you have any of the following symptoms, please enter 1. If not, please enter 0.

Group I Group II 　　　 Group III

| No. | Symptoms | Score | No. | Symptoms | Score | No. | Symptoms | Score |
| --- | --- | --- | --- | --- | --- | --- | --- | --- |
| 1 | My head is heavy | 1 or 0 | 11 | I can't think straight | 1 or 0 | 21 | I have a headache | 1 or 0 |
| 2 | My whole body feels tired | 1 or 0 | 12 | I don't like talking | 1 or 0 | 22 | My shoulders are stiff | 1 or 0 |
| 3 | My legs feel tired | 1 or 0 | 13 | Frustrating | 1 or 0 | 23 | Lower back pain | 1 or 0 |
| 4 | I yawn | 1 or 0 | 14 | Make more mistakes in doing | 1 or 0 | 24 | It's hard to breathe | 1 or 0 |
| 5 | My brain is foggy | 1 or 0 | 15 | Distracted | 1 or 0 | 25 | Thirsty | 1 or 0 |
| 6 | Sleepy | 1 or 0 | 16 | I can't be enthusiastic about things | 1 or 0 | 26 | My voice becomes hoarse | 1 or 0 |
| 7 | Eyes get tired | 1 or 0 | 17 | I can't remember little things | 1 or 0 | 27 | Feel dizzy | 1 or 0 |
| 8 | Movement is clumsy | 1 or 0 | 18 | I care about things | 1 or 0 | 28 | Eyelids and muscles twitch | 1 or 0 |
| 9 | I can't rely on my feet | 1 or 0 | 19 | I can't stay tidy | 1 or 0 | 29 | My limbs tremble | 1 or 0 |
| 10 | I want to lie down | 1 or 0 | 20 | I'm running out of patience | 1 or 0 | 30 | I don't feel well | 1 or 0 |

Group I evaluates drowsiness and lethargy (reduced vitality), Group II evaluates difficulty in concentrating (reduced energy), and Group III evaluates physical discomfort on the scale of fatigue symptoms.

How many hours did you sleep last night?
